# Prognostic significance of preoperative aspartate aminotransferase to neutrophil ratio index in patients with hepatocellular carcinoma after hepatic resection

**DOI:** 10.18632/oncotarget.10848

**Published:** 2016-07-26

**Authors:** Fei Ji, Shunjun Fu, Zhiyong Guo, Hui Pang, Dubo Chen, Xiaoping Wang, Weiqiang Ju, Dongping Wang, Xiaoshun He, Yunpeng Hua, Baogang Peng

**Affiliations:** ^1^ Organ Transplant Center, The First Affiliated Hospital, Sun Yat-sen University, Guangzhou, P. R. China; ^2^ Guangdong Provincial Key Laboratory of Organ Donation and Transplant Immunology, Guangzhou, P. R. China; ^3^ Guangdong Provincial International Cooperation Base of Science and Technology (Organ Transplantation), Guangzhou, P. R. China; ^4^ Department of Medical Records, The First Affiliated Hospital, Sun Yat-sen University, Guangzhou, P. R. China; ^5^ Department of Laboratory Medicine, The First Affiliated Hospital, Sun Yat-sen University, Guangzhou, P. R. China; ^6^ Department of Liver Surgery, The First Affiliated Hospital, Sun Yat-sen University, Guangzhou, P. R. China

**Keywords:** aspartate aminotransferase to neutrophil ratio index, systemic immune-inflammation, hepatocellular carcinoma, prognosis, biomarkers

## Abstract

**Objectives:**

Various inflammation-based prognostic scores have been associated with poor survival in patients with hepatocellular carcinoma (HCC), and neutrophils display important roles. However, few studies have illuminated the relationship between preoperative aspartate aminotransferase (AST) to neutrophil ratio index (ANRI) and poor prognosis of HCC. We aimed to clarify the prognostic value of ANRI and evaluate the ability of different inflammation-based prognostic scores such as ANRI, AST to lymphocyte ratio index (ALRI), AST to platelet count ratio index (APRI), neutrophil-lymphocyte ratio index (NLR), and platelet-lymphocyte ratio index (PLR).

**Methods:**

Data were collected retrospectively from 303 patients who underwent curative resection for HCC. Preoperative ANRI, ALRI, APRI, NLR, PLR and clinico-pathological variables were analyzed. Univariate, multivariate and Kaplan-Meier analyses were performed to identify the predictive value of the above factors for disease-free survival (DFS) and overall survival (OS).

**Results:**

ANRI was correlated with presence of HBsAg, AST, presence of cirrhosis, tumor size, PVTT, cancer of the liver Italian program (CLIP) score, recurrence. Univariate analysis showed ANRI, ALRI, APRI, NLR, PLR were significantly associated with DFS and OS in HCC patients with curative resection. After multivariate analysis, ANRI was demonstrated to be superior to ALRI, APRI, NLR, PLR, which were independently correlated with DFS and OS. Survival analysis showed that preoperative ANRI > 7.8 predicted poor prognosis of patients with HCC after hepatectomy. preoperative ANRI also showed different prognostic value in various subgroups of HCC. Furthermore, the predictive range was expanded by the combination of ANRI and NLR.

**Conclusions:**

preoperative ANRI is an independent effective predictor of prognosis for patients with HCC, higher levels of ANRI predict poorer outcomes and the combining ANRI and NLR increases the prognostic accuracy of testing.

## INTRODUCTION

Hepatocellular cancer (HCC) is one of the most common, and most aggressive malignancies, the third leading cause of cancer-related deaths worldwide [1, 2]. Although the substantial increase in diagnostic techniques and surgery, the long-term prognosis of patients still remained far from satisfactory because almost 70% of patients develop recurrence or metastasis within 5 years after surgery [3–5]. The absence of an accurate and sensitive clinical parameter has a profound effect on HCC therapeutic practice. It is therefore very important and urgent to search for an effective preoperative biomarker to identify patients with a high risk of recurrence or metastases, and provide personalized therapy to improve the clinical outcomes.

Aspartate aminotransferase (AST) is an enzyme reflecting the damage of liver. As a routine liver function index, it is always used to evaluate the progression of diseases concerning liver [6–8]. Witjes [9] has also reported that the high serum levels of AST may indicate a poor survival in HCC patients. Another factor affects the prognosis is the host's inflammatory response to cancer and tumor-mediated systemic inflammation [10]. It is now believed that inflammatory cells in the tumor microenvironment have significant effects on tumor development [11–13]. The elevation of pretreatment neutrophil, monocyte or leucocyte count has been associated with poor survival in various cancers including HCC [15–17]. Emerging evidence indicates that neutrophil may reflect the patients' inflammatory status and down-regulate host cellular immunity against cancer, thereby affecting the prognosis [14].

To our knowledge, the preoperative AST to neutrophil ratio index (ANRI) has not been conducted to predict the tumor recurrence and survival after curative resection for HCC, though preoperative AST to lymphocyte ratio index (ALRI) and AST to platelet count ratio index (APRI) have been demonstrated to be associated to the poor survival [17–19]. The aim of this study is to clarify the prognostic value of ANRI in HCC, we also evaluate the prognostic ability of different inflammation-based prognostic scores such as ANRI, ALRI, APRI, neutrophil-lymphocyte ratio (NLR), and platelet-lymphocyte ratio (PLR) to determine whether the ANRI could be a useful marker in predicting patients' outcomes.

## RESULTS

### Patient and tumor characteristics

The study included 271 male patients (89.4%) and 32 female patients (10.6%). The mean age was 50 years (range 21-79 years). A total of 222 patients (73.3%) developed recurrence and 192 patients (63.4%) died during follow up. Hepatitis B surface antigen (HBsAg) was positive in 266 patients (87.8%) and cirrhosis in 237 (78.2%) patients. Increased AFP levels (≥ 200 μg/L) were observed in 171 patients (56.4%), and 88 patients (29.0%) had multiple tumor masses. Mean tumor size was 80.7 mm (range 15-300 mm) in greatest diameter, and 198 (65.3%) patients had tumor ≥ 50 mm in diameter. According to the Edmonson-Steiner stage [20] of tumor differentiation, 235 (77.6%) patients were in stages I-II and 68 (22.4%) patients were in stages III-IV. Likewise, according to TNM classification [21], 175 (57.8%) patients were in TNM stage I and 128 (42.2%) patients in TNM stage II-III (Table 1).

**Table 1:** Correlation between preoperative ANRI and clinicopathologic characteristics in HCC

Variables	Cases	ANRI		*Χ*^2^	*r*	*P* value
		≤ 7.8	> 7.8			
Age(yrs)						
≥ 60	73	24(32.9%)	49(67.1%)	0.287		0.592
< 60	230	68(29.6%)	162(70.4%)			
Gender						
Male	271	80(29.5%)	191(70.5%)	0.862		0.353
Female	32	12(37.5%)	20(62.5%)			
HCC family history						
Yes	21	9(42.9%)	12(57.1%)	1.666		0.197
No	282	83(29.4%)	199(70.6%)			
HBsAg						
Positive	266	73(27.4%)	193(72.6%)	8.781	0.170	**0.003**
Negative	37	19(51.4%)	18(48.6%)			
AST(U/L)						
< 80	249	92(36.9%)	157(63.1%)	28.651	0.308	**<0.001**
≥ 80	54	0(0%)	54(100%)			
TBIL(μmol/L)						
< 34.2	277	88(31.8%)	189(68.2%)	3.018		0.082
≥ 34.2	26	4(15.4%)	22(84.6%)			
PLT(×10^9^)						
≥ 100	275	87(31.6%)	188(68.4%)	2.282		0.131
< 100	28	5(17.9%)	23(82.1%)			
Cirrhosis						
Yes	237	61(25.7%)	176(74.3%)	11.006	0.191	**0.001**
No	66	31(47.0%)	35(53.0%)			
AFP(μg/L)						
≥ 200	171	48(28.1%)	123(71.9%)	0.976		0.323
< 200	132	44(33.3%)	88(66.7%)			
Tumor size(cm)						
> 5	198	51(25.8%)	147(74.2%)	5.732	0.156	**0.017**
≤ 5	105	41(39.0%)	64(61.0%)			
Tumor number						
Single	215	71(33.0%)	144(67.0%)	2.478		0.115
Multiple	88	21(23.9%)	67(76.1%)			
TNM						
I	175	59(33.7%)	116(66.3%)	2.200		0.138
II-III	128	33(25.8%)	95(74.2%)			
Differentiation						
I-II	235	75(31.9%)	160(68.1%)	1.193		0.275
III-IV	68	17(25.0%)	51(75.0%)			
PVTT						
Yes	53	10(18.9%)	43(81.1%)	4.014	0.115	**0.045**
No	250	82(32.8%)	168(67.2%)			
CLIP score						
0-2	217	78(35.9%)	139(64.1%)	11.265	0.193	0.001
3-5	86	14(16.3%)	72(83.7%)			
Recurrence						
Yes	222	52 (23.4%)	170(76.6%)	18.915	0.250	**<0.001**
No	81	40(49.4%)	41(50.6%)			
Complication						
No	258	82(31.8%)	176(68.2%)	1.656		0.198
Yes	45	10(22.2%)	35(77.8%)			

HBsAg: hepatitis B surface antigen; AST: aspartate aminotransferase ratio index; TBIL: total bilirubin; PLT: platelet; AFP:alpha-fetoprotein; TNM: Tumor Node Metastasis; PVTT: portal vein tumor thrombus; CLIP: cancer of the liver Italian program; ANRI, Aspartate aminotransferase/neutrophil count ratio index.

### Determination of cut-off value

**Figure 1:**
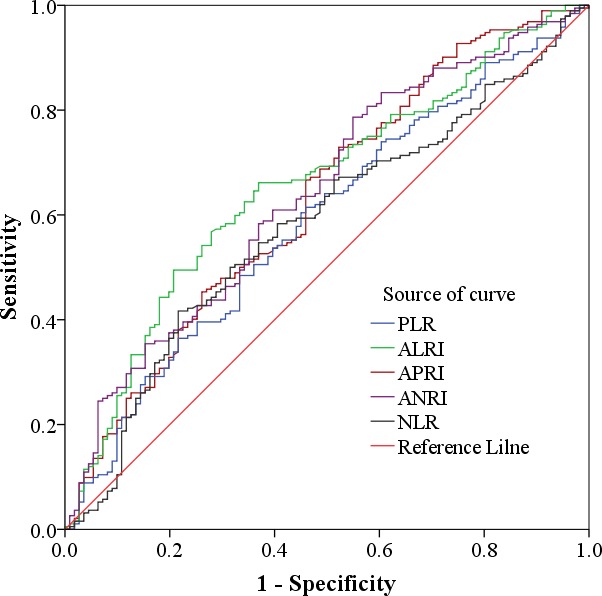
Determination of the cut-off value for NLR, PLR, ALRI, APRI, ANRI in HCC patients with hepatic resection

Using 5-year overall survival rate as an endpoint, stratification of NLR, PLR, ALRI, APRI, ANRI was calculated by ROC curve analyses. The results showed the Area Under the Curve (AUC) of NLR, PLR, ALRI, APRI, ANRI was 0.580, 0.588, 0.656, 0.629, 0.641 and the optimal cut-off value was 2, 115, 20, 1.68, 7.8, corresponding to the maximum joint sensitivity and specificity, respectively. (Figure 1).

### Correlation between ANRI and clinicopathologic characteristics in HCC

The relationship between preoperative ANRI and clinicopathological variables of patients with HCC was investigated, and the data showed that preoperative ANRI was correlated with HBsAg (*P* = 0.003), AST (*P* < 0.001), presence of cirrhosis (*P* = 0.001), tumor size (*P* = 0.006), PVTT (*P* = 0.045), cancer of the liver Italian program (CLIP) score (*P* = 0.001), recurrence (*P* < 0.001). the c-index between ANRI and CLIP were 0.70, which showed a concordance with the CLIP. Nevertheless, there were no significance between preoperative ANRI and other clinicopathological parameters such as age, gender, AFP, tumor number, TNM, tumor differentiation and complication (all *P* > 0.05, Table 1).

### Independent prognostic factors for HCC

In order to further identify the risk factors related to postoperative DFS and OS, ANRI and the clinicopathological parameters were evaluated by univariate analysis and the Cox regression model. The results revealed that preoperative ANRI (*P* < 0.001), HBsAg (*P* = 0.02), AFP (*P* < 0.001), AST (*P* < 0.001), Neutrophil (*P* = 0.018), CLIP score (*P* < 0.001), TNM (*P* = 0.001), PVTT (*P* < 0.001), tumor number (*P* < 0.001), tumor size (*P* < 0.001), tumor differentiation (*P* = 0.003), and intraoperative blood loss (*P* < 0.001), NLR (*P* < 0.001), APRI (*P* < 0.001), ALRI (*P* < 0.001), PLR (*P* = 0.025) were responsible for the DFS of HCC patients. Similarly, the significant predictors of OS in patients with HCC after resection were preoperative ANRI (*P* < 0.001), gender (*P* = 0.032), AFP (*P* = 0.006), AST (*P* < 0.001), Neutrophil (*P* = 0.032), CLIP score (*P* < 0.001), TNM (*P* < 0.001), PVTT (*P* < 0.001), tumor number (*P* < 0.001), tumor size (*P* < 0.001), tumor differentiation (*P* = 0.006), intraoperative blood loss (*P* < 0.001), NLR (*P* < 0.001), APRI (*P* < 0.001), ALRI (*P* < 0.001), PLR (*P* = 0.005) (Tables 2 and 3). After multivariate analysis, we found that AFP, tumor size, tumor number, PVTT, ANRI and Neutrophil were significant independent predictors of DFS (all *P* < 0.05), while tumor number, PVTT, ANRI, CLIP score, AST and NLR were significant independent predictors of OS (all *P* < 0.05) (Table 4).

**Table 2:** Prognostic factors for DFS and OS by univariate analysis

Variables	*n*	DFS			*P*	OS			*P*
		1-yr	3-yrs	5-yrs		1-yr	3-yrs	5-yrs	
Gender									
Male	271	43.2%	29.9%	26.0%	0.154	70.1%	44.6%	36.8%	**0.032**
Female	32	56.3%	37.5%	37.5%		81.3%	59.4%	56.3%	
Age(yrs)									
<60	230	43.9%	32.2%	29.5%	0.457	70.9%	47.4%	39.9%	0.430
≥60	73	46.6%	26.0%	20.0%		72.6%	42.5%	35.5%	
HBsAg									
Positive	266	41.4%	28.9%	25.0%	**0.02**	69.2%	44.4%	37.5%	0.075
Negative	37	67.6%	43.2%	43.2%		86.5%	59.5%	48.6%	
AFP(μg/L)									
< 200	132	54.5%	38.6%	36.3%	**< 0.001**	78.8%	53.0%	46.2%	**0.006**
≥200	171	36.8%	24.6%	20.3%		65.5%	40.9%	33.2%	
Hb(g/L)									
≤120	48	31.2%	16.7%	16.7%	0.052	68.7%	33.3%	29.2%	0.222
>120	255	47.1%	34.1%	29.2%		71.8%	48.6%	40.7%	
WBC(×10^9^)									
< 10	271	45.4%	31.4%	27.5%	0.588	72.3%	46.1%	39.0%	0.977
≥ 10	32	37.5%	25.0%	25.0%		62.5%	46.9%	37.5%	
AST(U/L)									
< 80	150	57.3%	41.3%	37.8%	**< 0.001**	83.3%	58.7%	50.6%	**< 0.001**
≥ 80	153	32.0%	20.3%	16.9%		59.5%	34.0%	27.4%	
Neutrophil(×10^9^)									
< 3.94	168	49.4%	35.1%	30.8%	0.018	78.6%	51.2%	44.0%	**0.032**
≥ 3.94	135	38.5%	25.2%	22.8%		62.2%	40.0%	32.6%	
CLIP score									
0-2	217	55.3%	39.2%	34.4%	**< 0.001**	80.6%	56.2%	48.8%	**< 0.001**
3-5	86	17.4%	9.3%	9.3%		47.7%	20.9%	13.4%	
TNM									
I	175	52.0%	36.6%	32.4%	0.001	77.7%	54.3%	47.3%	< 0.001
II-III	128	34.4%	22.7%	20.1%		62.5%	35.2%	27.3%	
Cirrhosis									
No	66	40.9%	27.3%	24.2%	0.454	75.8%	45.5%	37.9%	0.803
Yes	237	45.6%	31.6%	28.1%		70.0%	46.4%	39.2%	

HBsAg: hepatitis B surface antigen; AFP:alpha-fetoprotein; Hb: hemoglobin; WBC: white blood cell; AST: aspartate aminotransferase ratio index; CLIP: cancer of the liver Italian program; TNM: Tumor Node Metastasis.

**Table 3:** Prognostic factors for DFS and OS by univariate analysis

PVTT									
No	250	51.6%	35.2%	31.4%	**< 0.001**	78.8%	52.0%	44.7%	**< 0.001**
Yes	53	11.3%	9.4%	7.5%		35.8%	18.9%	11.0%	
Tumor number									
Single	215	53.5%	38.1%	33.9%	**< 0.001**	77.0%	54.9%	48.6%	**< 0.001**
Multiple	88	22.7%	12.4%	10.6%		56.8%	26.3%	16.7%	
Tumor size(cm)									
< 5	105	68.6%	51.4%	43.5%	**< 0.001**	88.6%	68.6%	60.0%	**< 0.001**
≥5	198	31.8%	19.7%	18.7%		62.1%	34.3%	27.7%	
Complication									
No	258	44.6%	32.2%	28.1%	0.424	72.5%	46.5%	40.2%	0.361
Yes	45	44.4%	22.2%	22.2%		64.4%	44.4%	30.8%	
Tumor differentiation									
I-II	235	48.5%	34.0%	30.5%	**0.003**	73.6%	48.9%	42.9%	**0.006**
III-IV	68	30.9%	19.1%	16.2%		63.2%	36.8%	24.8%	
Resection margin(cm)									
< 2	171	42.1%	26.9%	23.2%	0.186	70.8%	41.5%	34.4%	0.069
≥2	131	47.3%	35.1%	31.9%		71.8%	51.9%	44.2%	
Intraoperative blood loss(ml)									
≤ 1000	237	48.9%	34.6%	31.0%	**< 0.001**	76.4%	50.6%	43.8%	**< 0.001**
> 1000	66	28.8%	16.7%	13.6%		53.0%	30.3%	21.0%	
ANRI									
≤ 7.8	92	59.8%	46.7%	43.3%	**< 0.001**	81.5%	62.0%	55.4%	**< 0.001**
> 7.8	211	37.9%	23.7%	20.2%		66.8%	39.3%	31.6%	
NLR									
≤ 2	223	48.9%	35.9%	31.7%	**< 0.001**	75.3%	53.4%	45.7%	**< 0.001**
> 2	80	32.5%	16.2%	14.9%		60.0%	26.3%	20.0%	
APRI									
≤ 1.68	104	57.7%	41.3%	38.3%	**< 0.001**	82.7%	59.6%	52.9%	**< 0.001**
> 1.68	199	37.7%	25.1%	21.4%		65.3%	39.2%	31.5%	
PLR									
≤ 115	173	50.3%	35.8%	30.5%	**0.025**	75.7%	35.0%	45.6%	**0.005**
> 115	130	36.9%	23.8%	23.1%		65.4%	35.4%	29.9%	
ALRI									
≤ 20	135	57.0%	42.2%	38.5%	**< 0.001**	81.5%	60.7%	54.1%	**< 0.001**
> 20	168	34.5%	21.4%	18.0%		63.1%	34.5%	26.5%	

PVTT: portal vein tumor thrombus; ANRI, Aspartate aminotransferase/neutrophil count ratio index; NLR: neutrophil/lymphocyte ratio index; APRI: aspartate aminotransferase/platelet count ratio index; PLR: platelet/lymphocyte ratio index; ALRI: aspartate aminotransferase/Lymphocyte count ratio index.

**Table 4:** Independent prognostic factors for DFS and OS by the multivariate Cox proportional hazards regression model

*Variables*	*DFS*			*OS*		
	HR	95%CI	*P*	HR	95%CI	*P*
AFP	1.393	1.056-1.836	0.019			
Tumor Size	1.579	1.144-2.179	0.005			
Tumor number	0.581	0.435-0.777	< 0.001	0.650	0.465-0.910	0.012
PVTT	2.119	1.506-2.982	< 0.001	2.156	1.507-3.085	< 0.001
ANRI	1.747	1.222-2.498	0.002	1.617	1.108-2.358	0.013
Neutrophil	1.490	1.108-2.003	0.008			
CLIP score				1.459	1.015-2.097	0.042
AST				1.582	1.094-2.287	0.015
NLR				1.724	1.241-2.394	0.001

HR, hazard ratio; CI, confidence interval; AFP, Alpha-fetoprotein; PVTT, portal vein tumor thrombi; ANRI, Aspartate aminotransferase/neutrophil count ratio index; CLIP: cancer of the liver Italian program; AST: aspartate aminotransferase ratio index; NLR: neutrophil/lymphocyte ratio index.

### Overall and disease free survival rates according to ANRI

**Figure 2:**
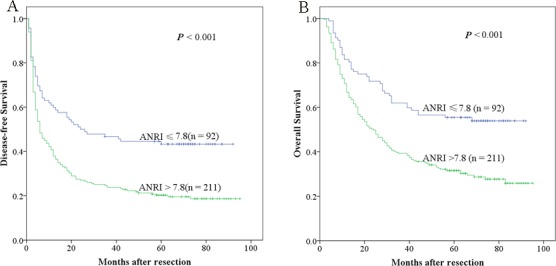
Relationship between ANRI and DFS/OS of HCC patients after hepatectomy **A.** DFS of patients with ANRI > 7.8 was significantly shorter than those with ANRI ≤ 7.8 (*P* < 0.001, log-rank test). **B.** OS of patients with ANRI > 7.8 was also markedly shorter than those with ANRI ≤ 7.8 (*P* < 0.001, log-rank test).

To determine the ability of ANRI to predict OS and DFS, the 303 HCC patients were divided into two groups: the ANRI ≤ 7.8 group (*n* = 92) and the ANRI > 7.8 group (*n* = 211). Using the Kaplan-Meier method to analyze patient survival, The data showed that the 1-, 3- and 5-year DFS rates of the ANRI ≤ 7.8 group were significantly higher than those of the ANRI > 7.8 group (59.8%, 46.7% and 43.3% *vs* 40.8%, 23.7% and 20.2%, respectively, *P* < 0.001) (Figure 2A), while the 1-, 3- and 5-year OS rates of the ANRI ≤ 7.8 group were also markedly higher than those of the ANRI > 7.8 group (81.5%, 62.0% and 55.4% *vs* 70.6%, 39.3% and 31.6%, respectively, *P* < 0.001) (Figure 2B). Therefore, our research displayed that preoperative ANRI > 7.8 levels were correlated with a poor survival.

### Prognostic values of preoperative ANRI in different HCC subgroups

**Figure 3:**
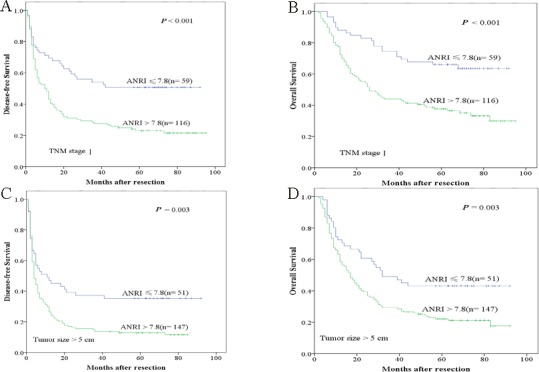
Kaplan-Meier survival curves of different HCC subgroups after hepatectomy ANRI > 7.8 significantly correlated with shorter DFS and OS in subgroups with TNM stage I **A.**, **B.** and tumor size > 5cm **C.**, **D.**

**Figure 4:**
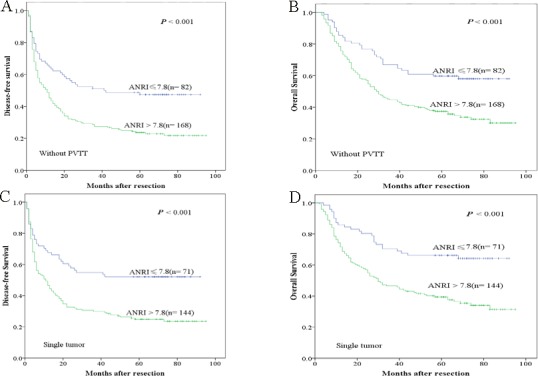
Kaplan-Meier survival curves of different HCC subgroups after hepatectomy ANRI > 7.8 significantly correlated with shorter DFS and OS in patients without PVTT **A.**, **B.** and with single tumor **C.**, **D.**

The research above verified that preoperative ANRI was an independent prognostic factor and significantly correlated with DFS and OS of HCC patients. We next assessed the prognostic value of preoperative ANRI in different subgroups of HCC patients. The results showed that preoperative ANRI was a prognostic indicator for DFS (69.5%, 54.2%, 50.7% *vs* 48.3%, 27.6%, 23.1%, *P* < 0.001, respectively) and OS (88.1%, 74.6%, 66.1% *vs* 77.6%, 44.0%, 37.7%, *P* < 0.001, respectively) in patients with TNM stage of I (Figure 3A, 3B). In addition, in the subgroup of tumor size > 5cm, preoperative ANRI > 7.8 also appeared noticeable prognostic value in predicting poorer DFS (47.1%, 37.3%, 35.3% *vs* 26.5%, 13.6%, 12.9%, *P* = 0.003, respectively) and OS (72.5%, 49.0%, 43.1% *vs* 58.5%, 29.3%, 22.2%, *P* = 0.003, respectively) (Figure 3C, 3D), and this prognostic value of DFS (64.6%, 51.2%, 47.4% *vs* 45.2%, 27.4%, 23.7%, *P* < 0.001, respectively) and OS (85.4%, 67.1%, 59.8% *vs* 75.6%, 44.6%, 37.3%, *P* < 0.001, respectively) also existed in patients without PVTT (Figure 4A, 4B) and in patients with single tumor (Figure 4C, 4D) (DFS: 67.6%, 54.9%, 52.1% *vs* 46.5%, 29.9%, 24.8%, *P* < 0.001, respectively; OS: 85.9%, 70.4%, 66.2% *vs* 71.5%, 46.5%, 39.4%, *P* < 0.001, respectively). These results further demonstrated that preoperative ANRI was more sensitive than other clinical parameters to predict the prognosis of HCC patients, especially in different kinds of HCC subgroups whose survival is too difficult to be predicted.

### The prognostic value of ANRI combined with NLR for HCC after hepatectomy

As both ANRI and NLR are neutrophil related factors, we decided to analyze the prognostic value for HCC survival of combining ANRI and NLR, the AUC value of the combination of the ANRI and NLR were 0.670, it is more than ANRI (the AUC is 0.641) and NLR (the AUC is 0.580), and significant difference was found between them (all *P* < 0.05). patients were divided into three groups: Group 1, NLR ≤ 2 and ANRI ≤ 7.8; Group 2, patients with NLR > 2 and ANRI ≤ 7.8 or with NLR ≤ 2 and ANRI > 7.8; Group 3, patients with both NLR > 2 and ANRI > 7.8.

**Figure 5:**
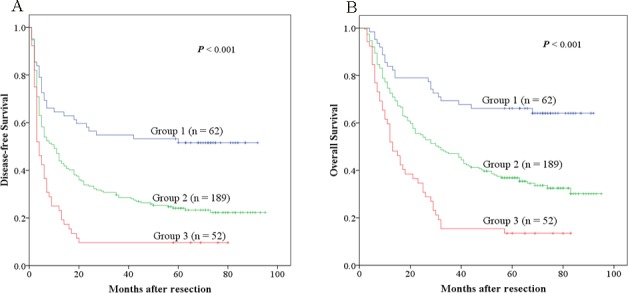
The combination of ANRI and NLR was found to enhance prognostic accuracy for HCC Disease-free survival curves **A.** and overall survival curves **B.** Group1: both ANRI ≤ 7.8 and NLR ≤ 2; Group2: both ANRI ≤ 7.8 and NLR > 2 or both ANRI > 7.8 and NLR ≤ 2; Group3: both ANRI > 7.8 and NLR > 2.

The 1-, 3- and 5-year DFS rates of Group 1 (64.5%, 54.8% and 51.6%, respectively) were significantly higher than those of Group 2 (43.9%, 28.6% and 24.1%, respectively, *P* < 0.001) and Group 3 (23.1%, 9.6% and 9.6%, respectively, *P* < 0.001). Similarly, the 1-, 3- and 5-year OS rates of Group 1 (83.9%, 69.4% and 66.1%, respectively) were also significantly higher than those of Group 2 (72.5%, 47.1% and 36.9%, respectively, *P* < 0.001) and Group 3 (51.9%, 15.4% and 13.5%, respectively, *P* < 0.001) (Figure 5A and 5B). Furthermore, we found that the 1-, 3- and 5-year DFS and OS rates of Group 2 were both significantly higher than those of Group 3 (*P* < 0.001 and *P* < 0.001) (Figure 5A and 5B).

## DISCUSSION

In order to improve the outcome of HCC patients, great effort had been made on searching for valid indicators to predict HCC prognosis. As most HCC was the result of chronic liver disease, the outcome of HCC may also depends on impaired liver function secondary to the underlying pathogenic condition, rather than solely the tumor burden. Tumor Node Metastasis (TNM), which includes only pathological variables, and shows the limited prognostic value in HCC. Therefore, A reliable prognostic index is needed for use in routine clinical practice.

Hematological components which reflect the systemic inflammatory response have been combined to construct the inflammation-based prognostic scores to predict survival. Neutrophil plays a central role in the systemic inflammatory response, and a number of studies have demonstrated a relation between the neutrophil count or neutrophil-lymphocyte ratio and the prognosis of cancer patients [22–25], one reason of the neutrophilia is the autocrine or paraneoplastic production of myeloid growth factors, Granulocyte-colony stimulating factor (G-CSF) is one of these factors which acts selectively on bone marrow granulocytic lineage cells resulting in granulopoiesis [26–28]. Another reason may be the cancer-related inflammation. Chronic inflammation can conduce to the initiation and progression of cancer. Because of the persistence of inflammation, the neutrophil also can directly down-regulate host cellular immunity against cancer, which affects the prognosis [14].

AST is a sensitive and reliable biochemical marker of liver injury, some liver diseases involving mitochondrial injury of hepatocytes, may lead the release of AST to serum. Witjes [9] had reported A higher AST level was showed to correlate with a greater influx of hepatitis B virus, which associates with decreased overall survival in HCC patients. Shen [19] and Jin [17] had certified that AST to platelet ratio index (APRI) and AST to lymphocyte ratio index (ALRI) were associated with a poor prognosis in HCC. So we aimed to demonstrate the preoperative ANRI might be a potential predictive marker for patients with HCC.

In our study, we first identified the cut-off value of preoperative ANRI according to the ROC curve, 7.8 appeared to be the optimal cut-off value of ANRI with a maximum joint sensitivity and specificity. Noteworthiness, concerning the correlationship between ANRI and clinical characteristics, we found that an elevated ANRI was positively related to HBsAg, AST, presence of cirrhosis, tumor size and CLIP score. what's more, the c-index between ANRI and CLIP were 0.70, which showed a concordance with the CLIP score. patients having an increased levels of ANRI are more inclined to have a higher rate of PVTT and recurrence. All these data certified that ANRI could not only reflect the liver injury induced by hepatitis B but also the tumor burden and tumor progression. Using univariate analysis, we discovered many significant prognostic factors for DFS or OS of HCC, including HBsAg, gender, AFP, AST, Neutrophil, CLIP score, TNM stages, PVTT, tumor size, tumor number, poor differentiation, intraoperative blood loss, NLR, PLR, ALRI, APRI and ANRI. However, After multivariate analysis, we catch that the independent related factors for both DFS were AFP, tumor size, tumor number, PVTT, ANRI and Neutrophil. while tumor number, PVTT, ANRI, CLIP score, AST and NLR were significant independent predictors of OS. Although the AUC of ALRI is larger than ANRI, there is no statistical significance between them (*P* = 0.591), So after multivariate analysis, ANRI was demonstrated to be superior to ALRI, APRI, NLR, PLR, which were independently correlated with DFS and OS

By a further analysis, we found that a shorter DFS and OS of HCC patients with preoperative ANRI > 7.8 had been documented in this study. The 1-, 3- and 5-year DFS rates and OS rates of patients with high level of ANRI were markedly lower than the low level group. Shau etc [29–31] had discovered that the neutrophils suppressed the cytolytic activity of lymphocytes and natural killer cells and the degree of suppression was in proportion to the number of neutrophils. CD4^+^ T lymphocyte cells act as a sensor in detecting precancerous cells and then regulate their eradication [32], these can prevent the occurrence and development of HCC. The loss of CD4^+^ T lymphocyte accompanied with the impaired activation of CD8^+^ T lymphocyte cells may cause the insufficient secretion of cytotoxin performing anti-carcinogenic function in the neoplastic microenvironments [33], what's more, Neutrophils potentiate cancer cell migration, invasion, and dissemination by secreting immunoreactive molecules such as hepatocyte growth factor (HGF) [34, 35], oncostatin M [36], b2-integrins [37] or neutrophil elastase [38]. With the progression of tumor diseases, the hepatic parenchymal cells are damaged, intracellular AST will be released into the blood which will cause a high concentration of serum AST. Therefore, the elevated preoperative ANRI indicates poorer prognosis of HCC.

In order to explicit the the prognostic value of preoperative ANRI in different subgroups of HCC patients, we found that preoperative ANRI had significant prognostic value for both DFS and OS in patients with TNM stage I, that is to say preoperative ANRI could be used to predict recurrence in early HCC. Besides, in subgroup with tumor size > 5 cm, patients with single tumor or patients without PVTT, preoperative ANRI > 7.8 also show its prognostic value in predicting poorer DFS and OS, all of these provided further evidence that preoperative ANRI can act as a potential prognostic marker to predict survival in HCC patients after hepatectomy.

Because both NLR and ANRI are prognostic factors relating neutrophil, we tried to explore whether the prognostic value could be expanded by the combination of them. Interestingly, our result exhibited that the combination of ANRI and NLR had a better prognostic value than either one alone, patients with NLR ≤ 2 and ANRI ≤ 7.8 had the best DFS and OS rates, patients with NLR > 2 and ANRI ≤ 7.8 or with NLR ≤ 2 and ANRI > 7.8 were the second, then patients with both NLR > 2 and ANRI > 7.8 were the worst.

As for other HCC biomarkers such as AFP, Glypican-3 (GPC3). the diagnostic sensitivity and accuracy haven't been conformed. Bialecki [39] had reported that 30% of HCC patients didn't have a remarkable rise of serum AFP, and AFP may also increase initially in the early stages of HCC but then drop or even normalize before rising again though disease progression occurs [40], Additionally, patients with chronic hepatitis and liver cirrhosis may have increased levels of AFP without HCC [41]. GPC3 is a member of the heparin sulfate proteoglycans, it can be secreted from HCC cells, However, it can be detected in only 40-53% of HCC patients and 33% of HCC patients seronegative for AFP [41–43]. Childs-pugh is a marker reflecting the liver function. For many HCC patients, their liver function is Childs pugh A, or liver function is normal. preoperative liver function may predict the short-term prognosis, but not for the long-term prognosis, because the liver function may change daily. And CLIP is a score combining factors of tumor and liver function, but it didn't display significance in DFS after multivariate analysis. So the ANRI not only meets the demands of highly precise diagnosis and prognosis, but also reduces patients' economic cost because it deduced from the routine examination, not extra examination. However, there are still some deficiencies in the present study. First, it is a single-institution, retrospective study. Second, our database mainly based on a cohort of HCC patients with predominant HBV infection in China. Therefore, the results for application in Western populations with predominant HCV infection or a history of alcohol abuse should be further confirmed.

In conclusion, our study demonstrate that the elevation of preoperative ANRI can be used as a prognostic factor for predicting the prognosis of patients with HCC after hepatectomy. These findings may suggest us to make the treatment plan considering not only TNM stage but also these prognosis­ related serum biomarkers. Only in this way can we acquire better personalised therapy for patients with HCC. In the future, this simple preoperative prognostic evaluation could be used to screen patients for personalized therapy.

## MATERIALS AND METHODS

### Ethics statement

Written informed consent was provided to all patients prior to surgery. Study approval was granted by the independent ethics committees at the First Affiliated Hospital of Sun Yat-sen University. This study was conducted in accordance with the ethical standards of the World Medical Association Declaration of Helsinki.

### Study population

A total of 303 histologically proven HCC patients with hepatic resection from our hospital were recruited between July 2006 and December 2009. Routine assessment was performed within 7 days before surgery, including a complete physical examination, hematologic and biochemistry profiles, chest X-ray, abdominal ultrasound and computed tomography (CT) or magnetic resonance imaging (MRI).

The eligibility criteria contained: the International Union Against Cancer (seventh edition) TNM stage I, II, IIIA or IIIB; Child-Pugh class A hepatic function; age 18-80 years. Exclusion criteria contained: TNM stage IIIC, IV; existing second malignancy or history of second malignancy within the past five years; hematologic disorders; systematic inflammatory diseases; perioperative dysfunction of vital organs; or percutaneous ablation, transcatheter arterial chemoembolization (TACE), chemotherapy or radiotherapy within one month after surgery.

### Treatment and follow-up

Hepatectomy was defined as radical when there was no evidence of distant metastases and tumor clearance was complete both macroscopically and histologically. All 303 patients were regularly followed up according to institutional practice, including liver ultrasound, chest X-ray and serum AFP every three months, and contrast CT every 6 months. Tumor relapse was diagnosed by clinical, radiological and/or pathological test. The mean for the postoperative follow-up period was 30.0 months (range, 2.0 to 95.0 months). Disease-free survival (DFS) was calculated from the date of surgery to the date of recurrence, and overall survival (OS) from the date of surgery to the date of HCC-associated death.

### Definition

Five inflammatory factors, including NLR, PLR, ALRI, APRI and ANRI were included in this analysis. The definitions of these factors are as follows: NLR = Neutrophil count/lymphocyte count; PLR = platelet count / lymphocyte count; ALRI = (AST value/lymphocyte count) × 10^9^/U; APRI = (AST value/platelet count) × 10^9^/U; ANRI = (AST value/Neutrophil count) × 10^9^/U. Postoperative complications were defined as occurrence of any medical or surgical complication during the hospital stay, such as Liver failure/insufficiency, Bile leak, Biloma/abscess, Intra-abdominal infection, Hemorrhage, Ascites, Wound infection/dehiscence, Pleural effusion, Pneumonia, Atelectasis, Arrhythmia, Heart failure, Urinary retention, Urinary tract infection, Renal insufficiency/failure, Ileus and Delayed Gastric Emptying.

### Statistical analysis

Statistical analysis was performed using SPSS for Windows version 20.0 (SPSS, Chicago, IL, USA). Receiver operating characteristic (ROC) curve analysis was performed to select the most appropriate cut-off values for ANRI to stratify patients at a high risk of death. The pROC package of R software (version 3.0.1) was used to evaluate the significance for the difference of area under the ROC curve (AUC). The χ^2^ test was used to compare categorical variables, and correlationship between variables were detected by Pearson test. The Kaplan-Meier method was used to estimate the survival rates for different groups, and the equivalences of the survival curves were tested by log-rank statistics. The Cox proportional hazards model was used for univariate and multivariate survival analyses. *P* < 0.05 was considered statistically significant.

## Abbreviations

HCC: hepatocellular cancer
TNM: Tumor Node Metastasis
NLR: neutrophil/lymphocyte ratio index
PLR: platelet/lymphocyte ratio index
AST: aspartate aminotransferase ratio index
ALRI: aspartate aminotransferase/lymphocyte count ratio index
APRI: aspartate aminotransferase/platelet count ratio index
ANRI: aspartate aminotransferase/neutrophil count ratio index
CLIP: cancer of the liver Italian program
GPC3: Glypican-3
CT: computed tomography
MRI: magnetic resonance imaging
TACE: transcatheter arterial chemoembolization
PLT: platelet
AFP: alpha-fetoprotein
ROC: receiver operating characteristics
DFS: disease-free survival
OS: overall survival
HBsAg: hepatitis B surface antigen
PVTT: portal vein tumor thrombus
TBIL: total bilirubin.
